# 3-Methyl-4-{(*E*)-[4-(methyl­sulfan­yl)benzyl­idene]amino}-1*H*-1,2,4-triazole-5(4*H*)-thione

**DOI:** 10.1107/S1600536813009690

**Published:** 2013-04-13

**Authors:** B. K. Sarojini, P. S. Manjula, Gurumurthy Hegde, Dalbir Kour, Vivek K. Gupta, Rajni Kant

**Affiliations:** a Department of Chemistry, PA College of Engineering, Nadupadavu 574 153, D.K. Mangalore, India; b Faculty of Industrial Science and Technology, University Malaysia Pahang, LebuhrayaTunRazak, 2630 0Gambang, Kuantan, Pahang Darul Makmur, Malaysia; c X-ray Crystallography Laboratory, Post-Graduate Department of Physics & Electronics, University of Jammu, Jammu Tawi 180 006, India

## Abstract

In the title mol­ecule, C_11_H_12_N_4_S_2_, the dihedral angle between the triazole and benzene rings is 21.31 (5)°. A weak intra­molecular C—H⋯S hydrogen bond generates an *S*(6) ring motif. In the crystal, pairs of N—H⋯S hydrogen bonds form inversion dimers. In addition, π–π inter­actions are observed between the benzene rings, with a centroid–centroid separation of 3.7599 (11) Å.

## Related literature

For background to Schiff base compounds, see: Dubey & Vaid (1991 ▶); Yadav *et al.* (1994 ▶); Galic *et al.* (2001 ▶). For biological applications of sulfur- and nitro­gen-containing compounds, see: Wei *et al.* (1981 ▶, 1982 ▶); Thieme *et al.* (1973*a* ▶,*b* ▶); Dornow *et al.* (1964 ▶); Barrera *et al.* (1985 ▶); Malik *et al.*, (2011 ▶). For related structures, see: Devarajegowda *et al.* (2012 ▶); Fun *et al.* (2008 ▶); Wang *et al.* (2008 ▶). For standard bond-length data, see: Allen *et al.* (1987 ▶). For hydrogen-bond graph-set motifs, see: Bernstein *et al.*(1995 ▶).
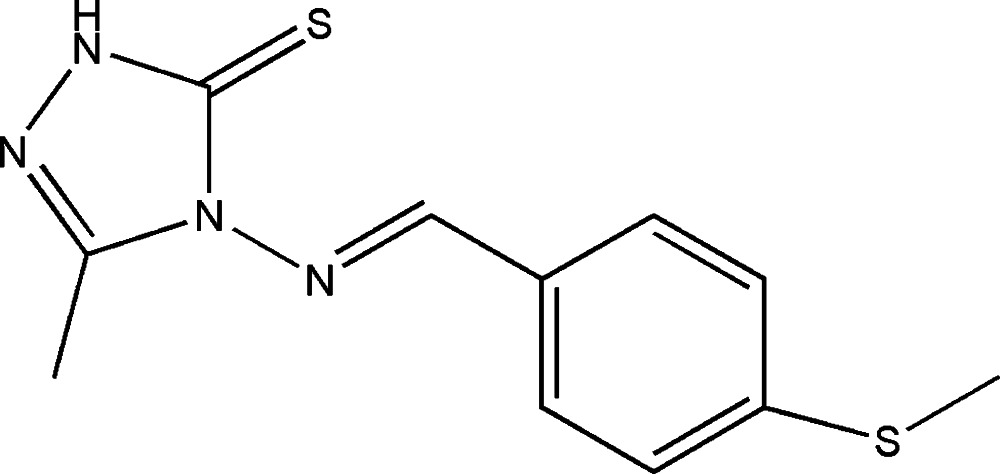


## Experimental

### 

#### Crystal data

C_11_H_12_N_4_S_2_
*M* *_r_* = 264.37
Triclinic, 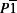

*a* = 7.7873 (2) Å
*b* = 9.5982 (2) Å
*c* = 9.6041 (2) Å
α = 76.608 (2)°
β = 70.602 (2)°
γ = 68.570 (2)°
*V* = 625.30 (2) Å^3^
*Z* = 2
Mo *K*α radiation
μ = 0.41 mm^−1^
*T* = 293 K
0.3 × 0.2 × 0.1 mm

#### Data collection

Oxford Diffraction Xcalibur Sapphire3 diffractometer
Absorption correction: multi-scan (*CrysAlis PRO*; Oxford Diffraction, 2010 ▶) *T*_min_ = 0.866, *T*_max_ = 1.000
30316 measured reflections
2449 independent reflections
2121 reflections with *I* > 2σ(*I*)
*R*_int_ = 0.041

#### Refinement

*R*[*F* ^2^ > 2σ(*F* ^2^)] = 0.032
*wR*(*F* ^2^) = 0.088
*S* = 1.03
2449 reflections
156 parameters
H-atom parameters constrained
Δρ_max_ = 0.26 e Å^−3^
Δρ_min_ = −0.17 e Å^−3^

### 

Data collection: *CrysAlis PRO* (Oxford Diffraction, 2010 ▶); cell refinement: *CrysAlis PRO*; data reduction: *CrysAlis PRO*; program(s) used to solve structure: *SHELXS97* (Sheldrick, 2008 ▶); program(s) used to refine structure: *SHELXL97* (Sheldrick, 2008 ▶); molecular graphics: *ORTEP-3 for Windows* (Farrugia, 2012 ▶) and *PLATON* (Spek, 2009 ▶); software used to prepare material for publication: *PLATON*.

## Supplementary Material

Crystal structure: contains datablock(s) I, global. DOI: 10.1107/S1600536813009690/lh5604sup1.cif

Structure factors: contains datablock(s) I. DOI: 10.1107/S1600536813009690/lh5604Isup2.hkl

Supplementary material file. DOI: 10.1107/S1600536813009690/lh5604Isup3.cml

Additional supplementary materials:  crystallographic information; 3D view; checkCIF report

## Figures and Tables

**Table 1:** Hydrogen-bond geometry (Å, °)

*D*—H⋯*A*	*D*—H	H⋯*A*	*D*⋯*A*	*D*—H⋯*A*
C8—H8⋯S1	0.93	2.57	3.212 (2)	126
N2—H2⋯S1^i^	0.86	2.48	3.328 (2)	169

Symmetry code: (i) 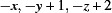.

**Fig. 1.**
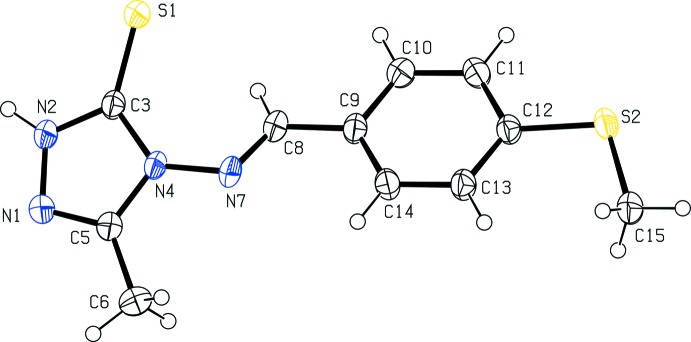
The molecular structure of the title compound with ellipsoids drawn at the 40% probability level. H atoms are shown as small spheres of arbitrary radii.

**Fig. 2.**
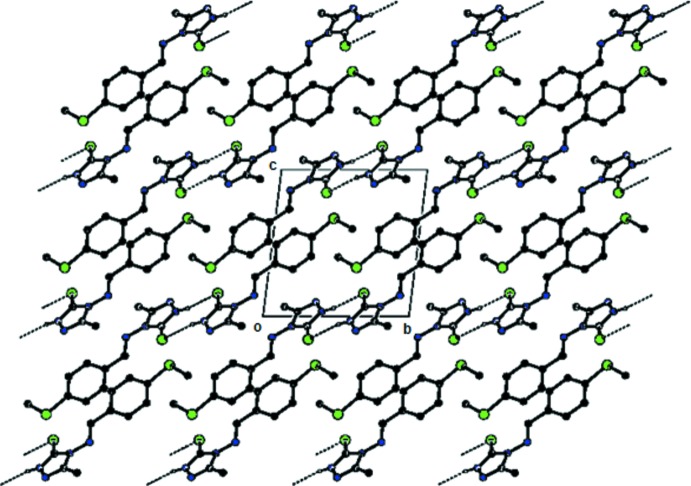
The packing arrangement of molecules viewed along the a axis. Hydrogen bonds are shown as dashed lines.

## supplementary crystallographic information

### Comment

During the last few decades, there has been a considerable interest in the chemistry of Schiff base compounds (Dubey & Vaid, 1991; Yadav *et al.*,1994). Schiff bases, containing different donor atoms, also find use in analytical applications and metal coordination (Galic *et al.*, 2001). Since many compounds containing sulfur and nitrogen atoms are antihypertensive (Wei *et al.*, 1981, 1982), analgesic (Thieme *et al.*, 1973*a*,*b*), anti-inflammatory (Dornow *et al.*, 1964), sedative (Barrera *et al.*, 1985), or fungicidal (Malik *et al.*, 2011), synthesis of the corresponding heterocyclic compounds could be of interest from the viewpoint of chemical reactivity and biological activity.

In the title compound (Fig. 1), the bond lengths and angles have values in the normal ranges (Allen *et al.*, 1987) and are comparable with closely related structures (Devarajegowda *et al.*, 2012; Fun *et al.*, 2008; Wang *et al.*, 2008). The dihedral angle between the triazole ring (N1/N2/C3/N4/C5) and the benzene ring (C9—C14) is 21.31 (5)°. The molecule exists in the thione tautomeric form, with an S═C distance of 1.681 (3) Å, which indicates substantial double-bond character for this bond [1.671 (24) Å](Allen *et al.*, 1987). The methylidene amino linkage (N7/C8) is slightly twisted from the mean plane of the 1,2,4 triazole ring as indicated by the torsion angle C3–N4–N7–C8 of -30.8 (2)°. A weak intramolecular C—H···S hydrogen bond generates an S(6) ring motif (Bernstein *et al.*,1995). The packing of molecules within the unit cell is shown in Fig. 2. In the crystal, pairs of N—H···S hydrogen bonds form inversion dimers. In addition, π–π interactions are observed between the benzene rings with Cg···Cg(-x,-y,1-z) = 3.7599 (11) Å, where Cg is the centroid of the C9—C14 ring.

### Experimental

To a suspension of 4-(methylthio)benzaldehyde (1.52 g, 0.01 mol) in methanol (15 ml), 4-amino-5-methyl-2,4-dihydro-3*H*-1,2,4-triazole-3-thione (0.01 mol, 1.65 g) was added and heated to form a clear solution. To this a few drops of conc.H_2_SO_4_ was added as a catalyst and refluxed for 5 h on a water bath. The precipitate formed was filtered and recrystallized from mixture of methanol and dioxane (2:1) to yield the titled compound. The single crystals were grown from a solution of the titlr compound of methanol (mp. 355–357 K).

### Refinement

All H atoms were positioned geometrically and were treated as riding on their parent C/N atoms, with C—H distances of 0.93–0.96 Å and N—H distance of 0.86 Å; and with *U*_iso_(H) = 1.2*U*_eq_(C/N), except for the methyl groups where *U*_iso_(H) = 1.5*U*_eq_(C),.

### Crystal data

**Table:**

C_11_H_12_N_4_S_2_	*Z* = 2
*M**_r_* = 264.37	*F*(000) = 276
Triclinic, *P*1	*D*_x_ = 1.404 Mg m^−^^3^
Hall symbol: -P 1	Mo *K*α radiation, λ = 0.71073 Å
*a* = 7.7873 (2) Å	Cell parameters from 15170 reflections
*b* = 9.5982 (2) Å	θ = 3.4–29.1°
*c* = 9.6041 (2) Å	µ = 0.41 mm^−^^1^
α = 76.608 (2)°	*T* = 293 K
β = 70.602 (2)°	Block, white
γ = 68.570 (2)°	0.3 × 0.2 × 0.1 mm
*V* = 625.30 (2) Å^3^	

### Data collection

**Table:**

Oxford Diffraction Xcalibur Sapphire3 diffractometer	2449 independent reflections
Radiation source: fine-focus sealed tube	2121 reflections with *I* > 2σ(*I*)
Graphite monochromator	*R*_int_ = 0.041
Detector resolution: 16.1049 pixels mm^-1^	θ_max_ = 26.0°, θ_min_ = 3.4°
ω scan	*h* = −9→9
Absorption correction: multi-scan (*CrysAlis PRO*; Oxford Diffraction, 2010)	*k* = −11→11
*T*_min_ = 0.866, *T*_max_ = 1.000	*l* = −11→11
30316 measured reflections	

### Refinement

**Table:**

Refinement on *F*^2^	Primary atom site location: structure-invariant direct methods
Least-squares matrix: full	Secondary atom site location: difference Fourier map
*R*[*F*^2^ > 2σ(*F*^2^)] = 0.032	Hydrogen site location: inferred from neighbouring sites
*wR*(*F*^2^) = 0.088	H-atom parameters constrained
*S* = 1.03	*w* = 1/[σ^2^(*F*_o_^2^) + (0.0446*P*)^2^ + 0.2502*P*] where *P* = (*F*_o_^2^ + 2*F*_c_^2^)/3
2449 reflections	(Δ/σ)_max_ = 0.001
156 parameters	Δρ_max_ = 0.26 e Å^−^^3^
0 restraints	Δρ_min_ = −0.17 e Å^−^^3^

### Special details

**Table:**

Experimental. *CrysAlis PRO*, Oxford Diffraction Ltd., Version 1.171.34.40 (release 27–08-2010 CrysAlis171. NET) (compiled Aug 27 2010,11:50:40) Empirical absorption correction using spherical harmonics, implemented in SCALE3 ABSPACK scaling algorithm.
Geometry. All e.s.d.'s (except the e.s.d. in the dihedral angle between two l.s. planes) are estimated using the full covariance matrix. The cell e.s.d.'s are taken into account individually in the estimation of e.s.d.'s in distances, angles and torsion angles; correlations between e.s.d.'s in cell parameters are only used when they are defined by crystal symmetry. An approximate (isotropic) treatment of cell e.s.d.'s is used for estimating e.s.d.'s involving l.s. planes.
Refinement. Refinement of *F*^2^ against ALL reflections. The weighted *R*-factor *wR* and goodness of fit *S* are based on *F*^2^, conventional *R*-factors *R* are based on *F*, with *F* set to zero for negative *F*^2^. The threshold expression of *F*^2^ > σ(*F*^2^) is used only for calculating *R*-factors(gt) *etc*. and is not relevant to the choice of reflections for refinement. *R*-factors based on *F*^2^ are statistically about twice as large as those based on *F*, and *R*- factors based on ALL data will be even larger.

### Fractional atomic coordinates and isotropic or equivalent isotropic displacement parameters (Å^2^)

**Table:**

	*x*	*y*	*z*	*U*_iso_*/*U*_eq_	
S1	0.15017 (7)	0.32917 (5)	0.84285 (5)	0.04691 (16)	
S2	0.40556 (6)	−0.40134 (5)	0.33254 (5)	0.03932 (14)	
N1	−0.3639 (2)	0.35160 (17)	1.09693 (16)	0.0409 (4)	
N2	−0.2053 (2)	0.39804 (16)	1.03276 (16)	0.0389 (3)	
H2	−0.1954	0.4764	1.0542	0.047*	
N4	−0.14673 (19)	0.20528 (14)	0.93293 (14)	0.0308 (3)	
N7	−0.0745 (2)	0.07943 (15)	0.85714 (15)	0.0356 (3)	
C3	−0.0676 (2)	0.31151 (18)	0.93426 (18)	0.0322 (3)	
C5	−0.3237 (2)	0.23338 (19)	1.03460 (18)	0.0351 (4)	
C6	−0.4492 (3)	0.1392 (2)	1.0656 (2)	0.0530 (5)	
H6A	−0.5573	0.1702	1.1492	0.079*	
H6B	−0.3784	0.0353	1.0876	0.079*	
H6C	−0.4932	0.1511	0.9800	0.079*	
C8	0.0461 (2)	0.08739 (18)	0.73055 (19)	0.0359 (4)	
H8	0.0818	0.1743	0.6941	0.043*	
C9	0.1295 (2)	−0.03748 (18)	0.64155 (18)	0.0332 (3)	
C10	0.2741 (3)	−0.0280 (2)	0.5103 (2)	0.0411 (4)	
H10	0.3162	0.0561	0.4831	0.049*	
C11	0.3554 (3)	−0.1409 (2)	0.4205 (2)	0.0413 (4)	
H11	0.4527	−0.1331	0.3339	0.050*	
C12	0.2930 (2)	−0.26694 (18)	0.45838 (18)	0.0324 (3)	
C13	0.1500 (2)	−0.27788 (19)	0.58982 (18)	0.0367 (4)	
H13	0.1084	−0.3622	0.6172	0.044*	
C14	0.0695 (2)	−0.16417 (19)	0.67990 (18)	0.0364 (4)	
H14	−0.0262	−0.1727	0.7674	0.044*	
C15	0.2776 (3)	−0.5350 (2)	0.4030 (2)	0.0513 (5)	
H15A	0.2824	−0.5749	0.5035	0.077*	
H15B	0.3350	−0.6157	0.3423	0.077*	
H15C	0.1464	−0.4864	0.4011	0.077*	

### Atomic displacement parameters (Å^2^)

**Table:**

	*U*^11^	*U*^22^	*U*^33^	*U*^12^	*U*^13^	*U*^23^
S1	0.0418 (3)	0.0503 (3)	0.0534 (3)	−0.0245 (2)	0.0056 (2)	−0.0266 (2)
S2	0.0395 (2)	0.0369 (2)	0.0370 (2)	−0.00991 (18)	0.00102 (18)	−0.01659 (18)
N1	0.0374 (8)	0.0447 (8)	0.0427 (8)	−0.0163 (7)	−0.0004 (6)	−0.0192 (7)
N2	0.0401 (8)	0.0359 (7)	0.0437 (8)	−0.0157 (6)	−0.0011 (6)	−0.0196 (6)
N4	0.0360 (7)	0.0278 (6)	0.0321 (7)	−0.0115 (5)	−0.0071 (6)	−0.0110 (5)
N7	0.0419 (8)	0.0298 (7)	0.0387 (8)	−0.0102 (6)	−0.0094 (6)	−0.0152 (6)
C3	0.0369 (8)	0.0293 (8)	0.0321 (8)	−0.0110 (7)	−0.0065 (7)	−0.0102 (6)
C5	0.0363 (8)	0.0378 (9)	0.0333 (8)	−0.0134 (7)	−0.0062 (7)	−0.0106 (7)
C6	0.0496 (11)	0.0582 (12)	0.0599 (12)	−0.0311 (10)	−0.0024 (9)	−0.0191 (10)
C8	0.0431 (9)	0.0310 (8)	0.0377 (9)	−0.0113 (7)	−0.0117 (7)	−0.0114 (7)
C9	0.0353 (8)	0.0314 (8)	0.0344 (8)	−0.0057 (7)	−0.0112 (7)	−0.0121 (7)
C10	0.0419 (9)	0.0377 (9)	0.0456 (10)	−0.0163 (8)	−0.0056 (8)	−0.0123 (8)
C11	0.0386 (9)	0.0414 (9)	0.0398 (9)	−0.0139 (8)	0.0018 (7)	−0.0135 (7)
C12	0.0310 (8)	0.0312 (8)	0.0331 (8)	−0.0038 (6)	−0.0087 (7)	−0.0103 (6)
C13	0.0414 (9)	0.0304 (8)	0.0369 (9)	−0.0112 (7)	−0.0049 (7)	−0.0104 (7)
C14	0.0389 (9)	0.0356 (9)	0.0316 (8)	−0.0098 (7)	−0.0034 (7)	−0.0107 (7)
C15	0.0498 (11)	0.0516 (11)	0.0546 (12)	−0.0224 (9)	0.0052 (9)	−0.0285 (9)

### Geometric parameters (Å, º)

**Table:**

S1—C3	1.6818 (17)	C8—C9	1.458 (2)
S2—C12	1.7565 (16)	C8—H8	0.9300
S2—C15	1.7837 (19)	C9—C14	1.388 (2)
N1—C5	1.294 (2)	C9—C10	1.395 (2)
N1—N2	1.3688 (19)	C10—C11	1.374 (2)
N2—C3	1.332 (2)	C10—H10	0.9300
N2—H2	0.8600	C11—C12	1.393 (2)
N4—C5	1.372 (2)	C11—H11	0.9300
N4—C3	1.3744 (19)	C12—C13	1.391 (2)
N4—N7	1.3944 (18)	C13—C14	1.382 (2)
N7—C8	1.272 (2)	C13—H13	0.9300
C5—C6	1.479 (2)	C14—H14	0.9300
C6—H6A	0.9600	C15—H15A	0.9600
C6—H6B	0.9600	C15—H15B	0.9600
C6—H6C	0.9600	C15—H15C	0.9600
			
C12—S2—C15	104.35 (8)	C14—C9—C10	118.42 (15)
C5—N1—N2	103.74 (13)	C14—C9—C8	123.14 (15)
C3—N2—N1	114.29 (13)	C10—C9—C8	118.44 (15)
C3—N2—H2	122.9	C11—C10—C9	121.01 (16)
N1—N2—H2	122.9	C11—C10—H10	119.5
C5—N4—C3	108.19 (13)	C9—C10—H10	119.5
C5—N4—N7	119.83 (13)	C10—C11—C12	120.40 (16)
C3—N4—N7	131.85 (13)	C10—C11—H11	119.8
C8—N7—N4	116.65 (13)	C12—C11—H11	119.8
N2—C3—N4	102.66 (14)	C13—C12—C11	118.93 (15)
N2—C3—S1	126.93 (12)	C13—C12—S2	125.20 (13)
N4—C3—S1	130.36 (12)	C11—C12—S2	115.88 (13)
N1—C5—N4	111.08 (14)	C14—C13—C12	120.34 (16)
N1—C5—C6	125.65 (16)	C14—C13—H13	119.8
N4—C5—C6	123.27 (15)	C12—C13—H13	119.8
C5—C6—H6A	109.5	C13—C14—C9	120.90 (16)
C5—C6—H6B	109.5	C13—C14—H14	119.6
H6A—C6—H6B	109.5	C9—C14—H14	119.6
C5—C6—H6C	109.5	S2—C15—H15A	109.5
H6A—C6—H6C	109.5	S2—C15—H15B	109.5
H6B—C6—H6C	109.5	H15A—C15—H15B	109.5
N7—C8—C9	121.10 (15)	S2—C15—H15C	109.5
N7—C8—H8	119.4	H15A—C15—H15C	109.5
C9—C8—H8	119.5	H15B—C15—H15C	109.5
			
C5—N1—N2—C3	−0.8 (2)	N4—N7—C8—C9	−179.55 (14)
C5—N4—N7—C8	153.95 (16)	N7—C8—C9—C14	7.1 (3)
C3—N4—N7—C8	−30.8 (2)	N7—C8—C9—C10	−174.05 (16)
N1—N2—C3—N4	1.71 (19)	C14—C9—C10—C11	0.1 (3)
N1—N2—C3—S1	−176.03 (13)	C8—C9—C10—C11	−178.77 (17)
C5—N4—C3—N2	−1.95 (17)	C9—C10—C11—C12	0.7 (3)
N7—N4—C3—N2	−177.60 (15)	C10—C11—C12—C13	−1.3 (3)
C5—N4—C3—S1	175.68 (14)	C10—C11—C12—S2	178.86 (14)
N7—N4—C3—S1	0.0 (3)	C15—S2—C12—C13	5.91 (18)
N2—N1—C5—N4	−0.59 (19)	C15—S2—C12—C11	−174.23 (14)
N2—N1—C5—C6	179.81 (18)	C11—C12—C13—C14	1.0 (3)
C3—N4—C5—N1	1.67 (19)	S2—C12—C13—C14	−179.17 (13)
N7—N4—C5—N1	177.93 (14)	C12—C13—C14—C9	−0.1 (3)
C3—N4—C5—C6	−178.72 (16)	C10—C9—C14—C13	−0.4 (3)
N7—N4—C5—C6	−2.5 (2)	C8—C9—C14—C13	178.42 (16)

### Hydrogen-bond geometry (Å, º)

**Table:**

*D*—H···*A*	*D*—H	H···*A*	*D*···*A*	*D*—H···*A*
C8—H8···S1	0.93	2.57	3.212 (2)	126
N2—H2···S1^i^	0.86	2.48	3.328 (2)	169

Symmetry code: (i) −*x*, −*y*+1, −*z*+2.
